# Development of a new tool for assessing Health-Related Quality of Life in patients with primary hyperparathyroidism

**DOI:** 10.1186/1477-7525-11-97

**Published:** 2013-06-18

**Authors:** Susan M Webb, Manel Puig-Domingo, Carles Villabona, Manuel Muñoz-Torres, Jordi Farrerons, Xavier Badia

**Affiliations:** 1 Department of Medicine/Endocrinology, Hospital Sant Pau, Pare Claret 167, Barcelona, 08025, Spain; 2 Centro de Investigación Biomédica de Enfermedades Raras (CIBER_ER Unit 747), Instituto de Salud Carlos III Universitat Autònoma de Barcelona, Barcelona, Spain; 3 Service of Endocrinology and Nutrition, Hospital Universitary Germans Trias i Pujol Crtra, Canyet, Badalona, 08916, Spain; 4 Service of Endocrinology and Nutrition, Hospital Universitary Bellvitge, C/ de la Feixa Llarga, L'Hospitalet de Llobregat, 08907, Spain; 5 Service of Endocrinology and Nutrition, Hospital Universitary San Cecilio, C/ Doctor Oloriz, 16, Granada, 18012, Spain; 6 Department of Medicine/Internal Medicine, Unidad de Metabolismo Minero-cálcico. Hospital Sant Pau, Pare Claret, 167, Barcelona, 08025, Spain; 7 IMS Health, C/ Dr. Ferrán, 25-27, 2nd floor, Barcelona, 08034, Spain

## Abstract

**Background:**

Several studies in recent years have evaluated Health Related Quality of Life (HRQoL) of patients with primary hyperparathyroidism (PHPT). No disease specific questionnaires are available to assess the impact of the disease. The aim of this research is to describe the development of a new disease specific Quality of Life (QoL) questionnaire for use specifically with PHPT patients.

**Methods:**

A conceptual model was developed describing the impact of the disease and its symptoms on QoL domains. A literature review was conducted to identify the most relevant domains. A focus group with experts was used to validate the domains; 24 patients were also interviewed to complement the information from the patient’s perspective. A content analysis of the interviews was performed to identify items related with the impact of the disease, leading to PHPQoL-V.1 which was presented to a sample of 67 patients. Reliability was assessed by Cronbach’s coefficient alpha and item-total score correlations. Validity was assessed by a factor analysis performed to determine the number of domains. Rasch analysis was carried out in order to refine the questionnaire items.

**Results:**

259 items were extracted from the interviews that were subsequently reduced to 34 items. Cronbach’s coefficient alpha was 0.92. The factor analysis extracted two domains (physical and emotional). After Rasch analysis the questionnaire PHPQoL-V.2 kept 16 items (9 physical and 7 emotional). The questionnaire was developed in a Spanish population and the final version was translated to English through translation and back-translation.

**Conclusion:**

The first disease specific HRQoL questionnaire for PHPT patients (PHPQoL-16) has been developed. Validation studies designed to assess measurement properties of this tool are currently underway.

## Introduction

Primary hyperparathyroidism (PHPT) is an endocrine disease characterized by hypercalcemia attributable to autonomous overproduction of parathyroid hormone (PTH). Therefore PHPT, is often detected by routine serum calcium measurement [1]. Poor control of elevated calcium levels can increase the risk of mortality due to cardiovascular, gastrointestinal, nervous, or kidney disorders or problems affecting bones and muscles [2]. In eight out of ten cases, the cause is a solitary, benign tumor (chiefcell adenoma) [3]. The prevalence of PHPT increases with age and is greater in women than in men; 80% of patients are apparently asymptomatic [4,5].

The diagnostic and therapeutic approaches to PHPT have changed in recent decades, but not without controversy. Prior to the 1970s, diagnosis was based purely on symptoms, meaning that the disease was only detected in symptomatic patients. The diagnosis of asymptomatic PHPT only became possible with the availability of biochemical tests that made it possible to test serum calcium levels and PTH [6]. Current PHPT treatment varies according to whether or not a patient has symptoms; this is reflected in different guidelines and consensus statements [7]. These guidelines suggest that all patients with confirmed PHPT and who present specific symptoms or signs of their disease should undergo surgical treatment. In addition, in some studies, surgical treatment has been associated with significant improvements in neuropsychological symptoms such as anxiety, depression, and mood swings [8-12], and HRQoL [13]. Improvement is particularly significant in patients with symptoms of depression and anxiety before surgery [14]. However, the benefit of surgery in terms of improving HRQoL in asymptomatic patients or in patients with minor symptoms is not so clear [15]. The most common pharmacologic treatment for apparently asymptomatic patients or patients who are not eligible for surgery are bisphosphonates, estrogens, vselective estrogen receptor modulators (SERMS), and calcimimetics [7].

Few studies have analyzed the impact of PHPT on HRQoL [16-19] and no disease specific questionnaires was available. A questionnaire specifically designed to assess the impact of symptoms on the emotional, social, and physical well being of PHPT patients would be useful to assess the impact of the disease and its treatment on patients’ HRQoL.

The aim of this study is to present the results of the development process of a disease specific HRQoL questionnaire for use in PHPT patients.

## Material and methods

**Figure 1:**
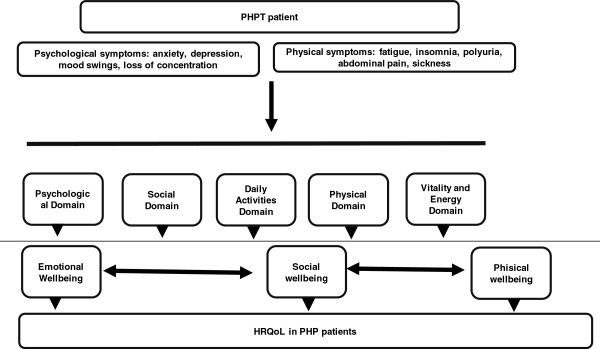
Conceptual framework.

According to FDA guidelines related to patient-reported outcome measures [20], the conceptual framework was established for the development of the questionnaire (Figure 1). A HRQoL specific questionnaire for PHPT should reflect the impact of PHPT on patients, and should be able to quantify the impact of physical and psychological symptoms. The ability of the questionnaire to reflect the impact of psychological symptoms was considered a key issue, as these symptoms are more difficult to identify by the endocrinologist, especially in patients who declare not to have any symptoms. In addition, it was also considered important to develop a questionnaire that took into account the relationship between symptoms and their impact on different domains of a patient’s life (psychological, social, physical, activities of daily living and energy/vitality). Considering all these aspects, the starting point was the development of a specific PHPT HRQoL questionnaire that would provide physicians with a tool to gain knowledge on the impact of PHPT on the patient’s daily life.

The HRQoL questionnaire for patients with PHPT was developed in three phases, namely generation, selection, and reduction of items [21-23].

### Item generation

The first step was to review studies and questionnaires dealing with HRQoL in PHPT included in MEDLINE in the past 10 years. The information obtained was used to identify topics and domains related to HRQoL and PHPT. A group of five experts (4 endocrinologists with experience in managing and treating PHPT patients, and one specialist in HRQoL questionnaires development) answered a semi-structured questionnaire in order to validate the information obtained from the literature review and identify those aspects they believed have an impact on patients’ life. Thereafter a Focus Group was organized with the same experts in order to reach a final consensus on the most important domains to be included in the final questionnaire. A Focus Group allows information to be collected through group interaction on a specific topic; giving rise to more information than that obtained from individual interviews [24].

### Item selection

In a second step, the resulting information on the most important domains identified by the experts was then used to create a semi-structured interview to identify which items had the greatest impact on patient’s daily life. A psychologist specializing in the development of patient-reported outcomes interviewed 24 patients with confirmed diagnosis of PHPT using this semi-structured interview. PHPT was diagnosed using clinical and biochemical data (hypercalcemia and elevated PTH after ruling out other causes of hypercalcemia). The interviews, which lasted between 30 and 45 minutes, were all audio-recorded and subsequently transcribed with the patients’ consent. The transcribed interviews were then used to extract items that indicated negative impact for each of the HRQoL domains analyzed.

An initial analysis and review of the content of the extracted items was performed by two psychologist to identify items that were considered inappropriate, redundant, or ambiguous. These items were removed from the item list. The remaining items were then reworded according to HRQoL expert opinion to produce a preliminary list of questionnaire items.

Each item was critically evaluated by the same five experts for clarity (ease of understanding by the patient), importance (relevance in terms of evaluating the impact of PHPT on HRQoL) and frequency (number of cases in which PHPT patients used the expression) [22,25,26]. The experts scored each item on a Likert-type scale ranging from 1 (not very clear/important/frequent) to 5 (very clear/important/frequent). Based on the scores assigned by the experts, items with a mean score of over 2.75 (25th percentile) were judged to be very clear; a score of over 2 was considered to be *very important* and *very frequent* (50th percentile in both cases). Items which did not comply with the following percentiles were removed: frequency × importance <50th percentile plus importance <50th percentile; frequency × importance <50th percentile plus frequency <50th percentile and clarity <25th percentile. In the case of clarity, items were removed when the problem was related to the concept rather than the wording. Other content analysis aspects were taken into consideration in the item reduction based on the judgment of three HRQoL experts.

### Item reduction

The identified items were then assembled into a questionnaire format (PHPQoL-V.1, with 34 items) (see ‘PHPQoL-V.1 (PHPQoL-34) subsection). A group of 67 active PHPT patients were asked to complete the PHPQoL-V.1 questionnaire (pilot study). This group of patients did not include the 24 patients who were interviewed previously. For each item, they were instructed to choose the option (always, often, sometimes, almost never, never) that better reflected their daily life. Data were also collected on age, gender, history of surgery for PHPT (yes/no) and kidney stone treatment, and fractures in the past two years.

### PHPQoL-V.1 (PHPQoL-34)

In the last month,…

1. I’ve felt tired or fatigued

2. I’ve felt sleepy after getting up in the morning and it’s been hard to get going

3. I’ve felt weak

4. I’ve found it difficult to walk for a long time

5. I’ve noticed that I get short of breath when I walk quickly

6. I’ve had difficulty going up and down stairs

7. I’ve been irritable

8. I’ve felt depressed

9. I’ve been down

10.  I’ve been sad

11.  I’ve been in a bad mood

12.  I’ve slept well

13.  I’ve woken up during the night

14.  I’ve had difficulty falling asleep

15.  I haven’t been able to remember things

16.  I’ve had difficulty remembering how to do routine activities (e.g., cooking)

17.  I’ve found it hard to concentrate

18.  I’ve been worried when I think about my illness

19.  I’ve been worried, not only about hyperparathyroidism but also its complications

20.  I’ve been worried that I might need an operation

21.  I’ve had kidney stones

22.  I’ve had stomach problems such as burning or gastritis

23.  I’ve been constipated

24.  I’ve had back pain

25.  My bones and/or joints have ached

26.  I’ve been able to carry out my activities as normal

27.  I’ve restricted some leisure activities because of the symptoms of the illness

28.  The illness has limited what household chores I do

29.  The illness has prevented me from making plans for the future (such as holidays,…)

30.  I’ve stopped doing things due to fear they would make my illness worse

31.  I’ve stopped doing things with friends because of my illness

32.  I’ve stopped working because of the illness

33.  The illness has prevented me from working

34.  I’ve noticed I find it more difficult to concentrate at work than before

Results of the pilot study were analyzed using Classical Test Theory (CTT) [27] and Item Response Theory (ITR) [28], and specifically on the Rasch analysis [29,30].

Using the CTT approach internal consistency (correlation of each item with total score, >0.7 theoretical) and reliability (Cronbach’s alpha, >0.7 theoretical) were assessed. Exploratory factor analysis was used to identify domains based on factor loadings. The total possible score on the PHPQoL-34 range from 34 (worst possible HRQoL) to 170 (best possible HRQoL). In order to facilitate interpretation of scores, these were normalized to a scale of 0 to 100, with higher scores indicating better HRQoL, using the following formula:

$$P_{100}=\left[100/\left(p_{\max}-p_{\min}\right)\right]*\left(p_{\mathrm{real}}-p_{\min}\right)$$

The Rasch model specifies that an individual’s answer to an item is the result of an interaction between the position of the item in the construct (calibration) and that of the individual (score). The model constructs a line in which each item is positioned and provides fit statistics to indicate how well an item describes the questionnaire’s answer. The analysis was performed using BIGSTEPS software, version 2.7.3. The term *measurement* is a measure of the difficulty of each item (a negative measurement indicates greater difficulty). The most common statistics used in this model are infit and outfit statistics (the information-weighted average of the squared standardized deviation of observed score with respect to expected score). Items with an infit or outfit mean-squares value of over 1.4 or less than 0.6 were excluded, as were items with a separation index of 1 for each domain [31]. Successive Rasch analyses were performed on each domain until all the items showed satisfactory fit.

The final Spanish version of the questionnaire was accurately translated into English following recommended standard methodology [32].

## Results

### Item generation

Our literature search retrieved 64 studies on HRQoL in PHPT; of these, 16 were selected for analysis of the most relevant items related to the impact of PHPT on daily life. The rest of studies were excluded because they were comparative studies of pharmacological treatments or because the measures used to evaluate HRQoL were not clear. Twenty-two items were chosen, including weakness, anxiety, depression, insomnia, apathy, lack of concentration, pain (muscle, bone, and joints), mood swings, loss of appetite, and digestive problems (vomiting, nausea). The different items were assigned to the following HRQoL domains: physical, emotional, social functioning, daily activities, symptoms, cognitive function, perceived health, sexual function, energy/vitality, and pain.

### Item selection

In total, 259 items considered to be relevant were extracted from the transcriptions of the interviews conducted in a group of 24 patients. Items considered to be redundant, ambiguous, or inappropriate were excluded. The number of items was reduced to 52 and reworded to form a suitable questionnaire items. These items were assessed by the group of experts, and those items that did not meet the criteria mentioned in the “Item Selection” section of “Material and Methods” were removed. The total number of items remaining after the qualitative analysis conducted by the HRQoL experts was 34.

### Item reduction

The PHPQoL-V.1 ( see ‘PHPQoL-V.1 (PHPQoL-34) subsection) was presented to a group of 67 PHPT patients (Table 1). Their mean (± SD) age was 59.2 (±13.4) years, 69.7% were women and 52.3% had a history of surgery for PHPT.

**Table 1:** Sociodemographic and clinical characteristics of the 67 patients who took part in the pilot studyof the PHPQoL-v.1 questionnaire

**Sociodemographic characteristics**	
Mean age (years), ±s.d.	59.2 ± 13.4
Female sex, n (%)	46 (69.7%)
**Clinical variables**	
Surgery (yes), n (%)	31 (47.7%)
No treatment for kidney stones, n (%)	49 (74.2%)
No bone fractures in the past two years, n (%)	65 (100%)*

* This percentage reflects all patients that answered this variable (65 of 67 patients).

The response rate was 80.9%. Most frequently unanswered items were 32, 33, and 34, all related to working life.

Internal consistency as measured by Cronbach’s alpha was 0.96 for the 34 items.

The mean normalized score obtained by the patients on the PHPQoL-V.1 was 63.2 ±19.70.

Those items not answered by over 50% of the patients (items 16, 29, 30, 31, 32, and 33 of PHPQoL-v1, see ‘PHPQoL-V.1 (PHPQoL-34) subsection) were eliminated. In total, six items were removed in this phase.

The remaining 27 items were subjected to exploratory factor analysis, which yielded two major domains explaining 51.5% of the total variance. The first domain explained 44.5% of the variance while the second domain explained 7%. All of the items that contributed to each of the domains had a factor loading of at least 0.4; item 10 *(I’ve been sad’)* had a similar loading on both factors but was included in the second one due to its similarity to the other items in this domain. None of the items in either of the two domains had a factor loading of less than 0.4 and were therefore not removed.

Thereafter, the Rash analysis was performed for each one of the domains. The Rasch model lists the items in relation to severity and selects the items according to adjustment to the model, redundancy, discriminant validity and content.

Following the application of Rasch analysis (Table 2), the number of items in the first domain was reduced from 12 to 11; item 6 (*I’ve had difficulty going up and down stairs*) was removed as it had an infit value of over 1.4. Overlapping items were assessed by the expert group with the aim of retaining just one item per level of impact. In the case of item 1 (*I’ve felt tired or fatigued*) and item 24 (*I’ve had back pain*), it was decided to retain item 24 as back pain is a common problem in PHPT and is also associated with a higher incidence of hip fracture [33,34]. Furthermore, the content of item 1 was covered by items 3 (*I’ve felt weak*) and 4 (*I’ve found it hard to walk for a long time*).

**Table 2:** Summary of Rasch analysis results for the physical and emotional functioning domains

**Physical functioning domain (factor 1) (PHPQoL-9)**					
**Item no.**	**Item**	**Measure**	**Error**	**Infit**	**Outfit**
2	I’ve felt sleepy after getting up in the morning and it’s been hard to get going	0.01	0.14	1.36	1.29
26	I’ve been able to carry out my activities as normal	−0.89	0.15	1.02	1.29
27	I’ve restricted some leisure activities because of the symptoms of the illness	−1.04	0.16	1.21	1.14
24	I’ve had back pain	0.72	0.14	1.19	1.18
5	I’ve noticed that I get short of breath when I walk quickly	−0.33	0.15	1.18	1.06
25	My bones and/or joints have ached	1.15	0.015	1.18	1.06
28	The illness has limited what household chores I do	−0.74	0.15	1.02	0.90
4	I’ve found it difficult to walk for a long time	0.25	0.14	0.88	0.89
3	I’ve felt weak	0.15	0.14	0.62	0.65
Emotional functioning domain (factor 2) (PHPQoL-7)					
Item no.	Item	Measure	Error	Infit	Outfit
17	I’ve found it hard to concentrate	−0.17	0.14	1.28	1.24
34	I’ve noticed I find it more difficult to concentrate at work than before	−0.73	0.16	1.19	1.04
12	I’ve slept well	−0.51	0.15	1.13	1.08
19	I’ve been worried, not only about the illness but also its complications	0.25	0.14	1.09	1.06
13	I’ve woken up during the night	0.80	0.14	0.85	0.90
8	I’ve felt depressed	0.15	0.14	0.84	0.83
7	I’ve been irritable	0.05	0.14	0.81	0.79

In the Rasch analysis of the second domain, five items were removed as they were outside the limits established for infit and outfit values. Items 7 (*I’ve been irritable*) and 14 (*I’ve had difficulty falling asleep*) overlapped and it was decided to retain item 7 as *irritability* had been mentioned frequently by the patients interviewed. Furthermore, sleep-related problems were already covered by items 12 and 13. Likewise, in the case of items 8 (*I’ve felt depressed*) and 18 (*I’ve been worried when I think about the illness*), it was decided to retain item 8 as the concept of worry was covered by item 19.

Item 16 was retained based on the results of the Rasch analysis but the option *not applicable* was added to the response categories, as the item was only applicable to patients who were working.

After applying Rasch analysis in each domain, the total number of items of the questionnaire was 16 (PHPQoL-V.2) (see ‘PHPQoL-V.2 (PHPQoL-16) subsection); 9 items corresponded to the first domain (PHPQoL-9) and 7 items to the second domain (PHPQoL-7). Based on the content of the items included in each domain, the first domain (PHPQoL-9) was labeled the physical functioning domain while the second one (PHPQoL-7) was labeled as the emotional functioning domain.

### PHPQoL-V.2 (PHPQoL-16)

Over the last 4 weeks and due to calcium problems, …

1. I've felt sleepy after getting up in the morning and it's been hard to get going

2. I've felt weak

3. I've found it hard to walk for a long time

4. I've noticed I get short of breath when I walk quickly

5. I’ve had back pain

6. My bones and/or joints have ached

7. I've found it difficult to carry out my daily activities

8. I've restricted some of my leisure activities

9. I've restricted what household chores I do

10.  I've been irritable

11.  I've felt depressed

12.  The illness has stopped me from sleeping well

13.  I've woken up during the night

14.  I've found it hard to concentrate

15.  I've been worried, not only about the illness but also its complications

16.  I've noticed I find it more difficult to concentrate at work than before

The correlations between the mean overall score obtained on the PHPQoL-16 and on each of the domains were over 0.90 and higher than the cutoff of 0.70. The correlation between the mean scores obtained on the PHPQoL-V.1 and the PHPQoL- V.2 was 0.98 (Table 3).

**Table 3:** Correlation between PHPQoL-V.2 (16 items), PHPQoL-9 (physical functioning), PHPQoL-7 (emotional functioning), and PHPQoL-V.2 (34 items) (version 1 of the questionnaire)

	**PHPQoL-V.2 (16 items)**	**PHPQol-9**	**PHPQoL-7**
PHPQol-9	0.96		
	P < 0.001		
	N = 61		
PHPQoL-7	0.90	0.76	
	P < 0.001	P < 0.001	
	N = 61	N = 61	
PHPQoL-V.1 (34 items)	0.98	0.93	0.91
	P < 0.001	P < 0.001	P < 0.001
	N = 55	N = 55	N = 55

Table 4 shows a summary of the most relevant results obtained for the initial 34- item (PHPQoL-V.1) and the reduced 16-item versions (PHPQoL-V.2).

**Table 4:** Summary of measurement properties of different versions of the PHPQoL questionnaire

		**PHP-QoL-V.2 (16 items)**	**PHPQoL-9**	**PHPQoL-7**	**PHPQoL-V.1 (34 items)**
			**(Physical functioning domain of V.2 of the PHPQoL questionnaire)**	**(Emotional functioning domain of V.2 of the PHPQoL questionnaire)**	**Piloted version of questionnaire**
No. of items		16	9	7	34
Score distribution	Valid observation	61	66	62	55
	Mean	58.73	58.08	59.10	63.2
	SD	21.95	25.49	20.82	19.70
	25th percentile	43.75	38.89	42.86	50.74
	50th percentile	57.81	55.56	60.71	66.18
	75th percentile	76.56	77.78	78.57	80.88
Total item correlation		0.45-0.81	0.66-0.84	0.57-0.77	0.03-0.81
Internal consistency (Cronbach’s alpha)		0.92	0.91	0.82	0.96
Rasch analysis					
Person separation		3.06	2.87	2.23	3.72
Person reliability		0.90	0.89	0.83	0.93

There were statistically significant differences between overall scores when analyzed by gender, with women scoring lower than men (p < 0.01). We also observed worse HRQoL with increasing age, but this did not reach statistical significance (p = 0.52). There were no significant differences in scores depending on whether or not the patients had undergone surgery (p = 0.074) or had been treated for kidney stones (p = 0.23). None of the patients reported fractures in the past 2 years.

The PHPQoL-v.2 was translated by two professional (bilingual) translators with experience in translating HRQOL questionnaires; in a consensus meeting both translations were compared with each other and with the original Spanish version. This version was then independently translated back into Spanish to ascertain equivalent significance in both languages.

## Discussion

We have developed a disease specific HRQoL questionnaire for PHPT patients using classical test theory and item response theory [22,23]. The first version composed of 34 items was reduced resulting in a final version of the question containing 16 items across two domains: physical and emotional. The results suggest the adequacy of the 16-item questionnaire for evaluating in a valid and reliable way HRQoL of patients with PHPT.

The PHPQoL questionnaire has been specifically designed to be used with those patients with PHPT. It is designed to quickly assess the impact of the emotional/neuropsychological and physical aspects of the disease that can highly affect patients’ lives, but that are difficult to quantify in a fast, standardized manner in routine clinical practice. Furthermore, PHPQoL has all the elements required to become a suitable questionnaire for both routine clinical practice and research: it has few items, is easily understandable by the patient, and contains short questions and response categories. The definitive scoring system and clinically relevant thresholds will be determined in the validation phase of the study, which is already underway. The current version of the PHPQoL also has good preliminary measurement properties.

Up to date, the impact of PHPT on patients’ HRQoL has been measured using generic questionnaires. The advantage of disease specific questionnaires versus generic ones is that the former allow assessing the impact of specific, often exclusive, aspects of a disease and its treatment on patient’s daily lives. While studies performed in PHPT patients with generic questionnaires, such as the SF-36 [13,15], have shown that surgery and other treatments improve HRQoL of symptomatic patients, particularly in aspects related to vitality, social functioning, general health, and mental health, the cognitive aspects related to memory loss or attention deficit cannot be evaluated using these instruments.

Other existing questionnaires are largely used in clinical practice to measure the severity of the symptoms, but these questionnaires do not link the severity of symptoms with their impact on a patient’s daily life. An example of this is the Pasieka’s parathyroid symptoms score, which evaluates the severity of PHPT symptoms and detects changes after surgery [35]. Other questionnaires are the Beck Depression Inventory which measures neuropsychological symptoms such as anxiety, depression, and memory problems, the Hamilton Anxiety and Depression scale [36], the North American Adult Reading Test [37], and the Rey Auditory Verbal Learning Test [38] to evaluate HRQoL, but none of these questionnaires is specific or was designed to evaluate PHPT patients.

One of the interests of those specialists who treat PHPT patients is the impact of their treatments on the HRQoL in symptomatic and apparently asymptomatic patients. The HRQoL described in this manuscript appears capable of serving that function. Up to date the majority of studies have concluded that both quality of life and neuropsychological symptoms improve after surgery [12,14,39], but HRQoL has been measured through generic questionnaires. The disease specific HRQoL PHPT questionnaire will be a useful tool in clinical practice to understand the impact of treatment on patients’ daily life.

Results of the pilot study (67 patients) showed no significant differences in scores depending on whether or not the patients had undergone surgery or had been treated for kidney stones, but the sample of patients treated with kidney stones was very small. It is to be expected that a larger sample of patients with kidney stones, would show differences between patients with and without symptoms in terms of HRQoL.

Only preliminary data are available on the measurement properties of the PHPQoL questionnaire. A validation study is currently underway involving a large sample of PHPT patients with the aim of determining the measurement properties of this questionnaire such as validity, reliability and sensitivity to change.

In conclusion, these results suggest that the described HRQoL questionnaire is useful for defining the impact of PHPT on the HRQoL of these patients.

## Competing interests

The authors do not have any conflict of interests.

## Authors’ contributions

All authors have contributed equally in the questionnaire & study development. All authors read and approved the final manuscript.
